# Absolute quantification of viable *Vibrio cholerae* in seawater samples using multiplex droplet digital PCR combined with propidium monoazide

**DOI:** 10.3389/fmicb.2023.1149981

**Published:** 2023-06-09

**Authors:** Jinsong Yang, Haibin Xu, Zili Ke, Naipeng Kan, Enhui Zheng, Yufeng Qiu, Mengying Huang

**Affiliations:** ^1^Fujian Center for Disease Control and Prevention, Fuzhou, China; ^2^Fujian Provincial Key Laboratory of Zoonosis Research, Fuzhou, China; ^3^Department of Preventive Medicine, School of Public Health, Fujian Medical University, Fuzhou, China

**Keywords:** *Vibrio cholerae*, droplet digital PCR, propidium monoazide, multiplex detection, seawater

## Abstract

**Introduction:**

Toxigenic *Vibrio cholerae* serogroup O1 and O139 are the pathogens responsible for the global cholera epidemic. *V. cholerae* can settle in the water and spread *via* the fecal-oral route. Rapid and accurate monitoring of live *V. cholerae* in environmental water has become an important strategy to prevent and control cholera transmission. Conventional plate counting is widely used to detect viable bacteria but requires time and effort.

**Methods:**

This study aims to develop a new assay that combines triplex droplet digital PCR (ddPCR) with propidium monoazide (PMA) treatment for quantitatively detecting live *V. cholerae* O1/O139 and cholera enterotoxin. Specific primers and probes were designed according to the conserved regions of gene *rfb* O1, *rfb* O139, and *ctx*A. The amplification procedures and PMA treatment conditions were optimized. The specificity, sensitivity, and ability of PMA-ddPCR to detect viable bacteria-derived DNA were evaluated in simulated seawater samples.

**Results and Discussion:**

The results revealed that the optimal primer concentrations of *rfb* O1, *rfb* O139, and *ctx*A were 1 μM, while the concentrations of the three probes were 0.25, 0.25, and 0.4 μM, respectively. The best annealing temperature was 58°C to obtain the most accurate results. The optimal strategy for distinguishing dead and live bacteria from PMA treatment was incubation at the concentration of 20 μM for 15 min, followed by exposure to a 650-W halogen lamp for 20 min. In pure culture solutions, the limit of detection (LODs) of *V. cholerae* O1 and O139, and *ctx*A were 127.91, 120.23 CFU/mL, and 1.5 copies/reaction in PMA-triplex ddPCR, respectively, while the LODs of the three targets were 150.66, 147.57 CFU/mL, and 2 copies/reaction in seawater samples. The PMA-ddPCR sensitivity was about 10 times higher than that of PMA-qPCR. When detecting spiked seawater samples with live bacterial concentrations of 1.53 × 10^2^ and 1.53 × 10^5^ CFU/mL, the assay presented a higher sensitivity (100%, 16/16) than qPCR (50.00%, 8/16) and a perfect specificity (100%, 9/9). These results indicate that the developed PMA-triplex ddPCR is superior to the qPCR regarding sensitivity and specificity and can be used to rapidly detect viable toxigenic *V. cholerae* O1 and O139 in suspicious seawater samples.

## Introduction

Cholera is a severe intestinal infectious disease caused by *Vibrio cholerae* and has resulted in seven pandemics worldwide (Deen et al., 2020). It is still a serious threat to global public health. Over the past few years, the number of cholera case reported to WHO has continued to be high. During 2020, 323,369 cases and 857 deaths were notified from 24 countries (World Health Organization, 2021). There are more than 200 serogroups of *V. cholerae*, but only O1 and O139 that produce Cholera enterotoxin (CT) can cause cholera epidemics in humans (Safa et al., 2010). *V. cholerae* is mainly transmitted by the fecal-oral route and can continuously contaminate water bodies. *V. cholerae* can survive in aquatic reservoirs in a variety of forms, they can also coexist with zooplankton, copepods, or other natural aquatic hosts. Toxigenic O1 and O139 are also the inhabitants in these environmental reservoirs (Colwell and Huq, 1994). A study investigating the phylogeography of both clinical and aquatic *V. cholerae* O1 isolates in Haiti have shown that aquatic reservoirs actively promote genetic diversification and the epidemic emergence (Mavian et al., 2020). Moreover, the increase in sea surface temperature and marine pollution in recent years have led to the population explosion and geographical diffusion of marine pathogens, such as *V. cholerae* of the *Vibrio* family, resulting in potential increases in *Vibrio*-related disease incidence and the spread of these infections to new and previously unaffected areas (Escobar et al., 2015). Climatic catastrophes may also increase the risk of the spread of pathogenic vibrio infections and exposure to disease by altering the geographic landscape and pathogen ecology through coastal flooding, seawater intrusion into inland and cyclones (Jutla et al., 2017; Froelich and Daines, 2020). Therefore, rapid and sensitive detection of *V. cholerae* O1/O139 and *ctx*A genes is crucial for early response and cholera epidemic control.

The traditional bacterial culture method is the “golden standard” for detecting *V. cholerae* (Alam et al., 2010; Ramamurthy et al., 2020). Although this method can obtain viable bacteria that can be proliferated for subsequent tests, the disadvantages are the time-consuming, laborious, low sensitivity, and susceptible to interference (Keddy et al., 2013). Additionally, *V. cholerae* can enter the viable but non-cultivable state (VBNC) in the environment as a survival strategy against sub-lethal stresses, such as extreme temperature, pH, and low nutrient concentrations, especially in aquatic ecosystems (Xu et al., 1982; Li et al., 2014). In this state, the strains retain their host’s infectious potential but temporarily lose their ability to grow on laboratory media (Cenciarini-Borde et al., 2009). Therefore, culture-based methods likely underestimate the risk of *V. cholerae* contamination. Recent molecular techniques, including nucleic acid amplification, have provided sensitive, rapid, and quantitative analytical tools for studying various pathogens to evaluate the microbial quality of water (Girones et al., 2010). Conventional Polymerase Chain Reaction (PCR) poorly distinguishes between DNA derived from live bacterial cells, inactivated cells, and fragments associated with free DNA (Cangelosi and Meschke, 2014). A viability PCR (v-PCR) strategy has been developed to overcome this difficulty that combines propidium iodide (PMA) dye with quantitative PCR (qPCR) to correlate viability with cell envelope impermeability. PMA is a photoactive DNA-binding dye that can easily penetrate dead bacteria through damaged cell membranes. When exposed to bright light, the azide group of PMA converts into the reactive nitro group, which further reacts with the hydrocarbon part of the DNA strand to form a strong nitro-carbon covalent bond. This modification results in structural changes and insolubility of DNA, causing DNA loss during extraction, and DNA polymerase cannot obtain DNA for amplification (Nocker et al., 2006; Askar et al., 2019). More researchers have explored real-time PCR combined with PMA for detecting viable bacteria (Nocker et al., 2007). However, only Wu (Wu et al., 2015) used PMA combined with qPCR to count *V. cholerae* O1 in the VBNC state, which still had limitations in simultaneous detection or differentiation between live *V. cholerae* O1 and O139 and understanding the carriage status of *ctx*A gene.

Droplet digital PCR (ddPCR), as the third-generation PCR technology, can directly determine the initial template copy number by microdroplet processing and distribution of the reaction system to an independent reaction chamber. The initial template copy number can be determined directly by reading the fluorescence signal in each reaction chamber and performing Poisson statistics. The absolute quantification can be achieved without a standard curve. ddPCR provides more sensitive, accurate, and reproducible data than qPCR. It exhibits greater tolerance to PCR-inhibiting substances typically present in environmental samples (Taylor et al., 2017). Since its commercialization in 2011, it has been widely employed in clinical and environmental studies (Cao et al., 2015; Chen et al., 2021). Recently, the multiplex ddPCR method has been developed based on single-target ddPCR that can detect multiple targets with high accuracy and sensitivity to reduce detection time and improve efficiency. Multiplex ddPCR has been used to quantitatively detect the contamination levels of *Shiga* toxin *Escherichia coli* (STEC), *Salmonella enterica*, and *Listeria monocytogenes* in surface water (Cooley et al., 2018), as well as simultaneous detection of five biothreatening pathogens in soil (Du et al., 2022). However, multiplex ddPCR has not been reported to detect toxigenic *V. cholerae* O1 and O139 simultaneously.

This study aims to establish a triplex ddPCR method combined with PMA treatment to simultaneously enumerate viable cells of *V. cholerae* O1/O139 accurately and sensitively, as well as evaluate its potential to produce cholera enterotoxin. The specificity, sensitivity, and detection ability of the method for the determination of toxigenic O1 and O139 *V. cholerae* live strains, as well as the detection performance in artificial seawater samples spiked with *V. cholerae* were comprehensively evaluated. This method can provide technical support for the detection, prevention and control of cholera epidemics.

## Materials and methods

### Bacterial culture and preparation of live/dead bacterial suspension

In this experiment, optimization and validation tests were performed by using 13 reference strains, eight clinical isolates and 10 environmental isolates (Supplementary Table S1). Serogroup O1, O139 and non-O1/non-O139 *V. cholerae* strains were identified and distinguished by automated identification system (Vitek 2 Compact, BioMerieux, France), serum agglutination test and PCR method, and whether the strains carried *ctx*A gene was confirmed by PCR. The strains were stored in 20% (v/v) glycerol at −80°C. All bacterial culture experiments were conducted in a Biosafety Level 2 laboratory. A single colony was isolated on nutrient agar medium and inoculated in Luria-Bertani (LB) broth (Oxoid, Basingstoke, UK) with shaking (180 rpm) at 37°C overnight. Briefly, 1 mL bacterial solution was centrifuged at 6,000 × g for 3 min, and the precipitate was washed with phosphate-buffered solution (PBS) twice. The precipitate was resuspended to 0.5 McFarland (MCF) (approximately 1.5 × 10^8^ CFU/mL) and quantified by plate counting using a bacterial turbidimeter (Densimat, Biomerieux, France). To confirm the accuracy of the method, the results obtained by plate counting were compared with those obtained by blood cell counting, which was performed by microscope to directly count bacteria cells suspended in the liquid (Thomson and Gunsch, 2015), and the difference between these two methods was not statistically significant.

The counted bacterial suspension was divided into two aliquots: a viable cell suspension and another incubated at 95°C for 15 min to obtain heat-killed cell suspension. Briefly, 500 μL heat-killed cell suspension was plated on nutrient agar in triplicate to evaluate the heat-killed effect. The absence of colony formation after 48 h of incubation at 37°C proved that *V. cholerae* had been killed.

### Genomic DNA extraction

The bacterial suspension (300 μL) was extracted using a bacterial genomic DNA extraction kit (Qiagen, Germany) according to the manufacturer’s instructions. The extracted genomic DNA was eluted with 70 μL Tris-EDTA (TE) buffer. DNA samples were quantified using a Qubit fluorescence quantizer (Thermofisher, USA) and stored at −20°C.

### Primers and probes

According to the published literature, *rfb* O1 (*rfb*N), *rfb* O139 (*wbf*R), and *ctx*A gene sequences were downloaded from GenBank National Center for Biotechnology Information (NCBI). After homology alignment, specific primers and probes (Table 1) were designed in the conserved regions of the respective genes using Primer Express 3.0.1 software. Sequence specificity of the primers and probes was assessed using the GenBank Primer-BLAST tool and synthesized by Sangon Biotech, Shanghai, China.

**Table 1:** Primer and probe sequences of the three target genes for detection.

Target gene	Sequence (5′ to 3′)	Reference Seq and Position (bp)	Product size (bp)
*rfb* O1	F: CTACCGCATTCATATCCG	X59554.1:13334–13,361	76
	R: CACACTCACAAAGACTTTC	X59554.1:13401–13,419	
	P: FAM-TCAATCACACCAAGGTCATCTGTAAGT-BHQ1	X59554.1:13373–13,399	
*rfb* O139	F: ACCTGTTATGTACGATGAA	AB012956.1:33624–33,642	98
	R: CAGACAAGCATACAGTGA	AB012956.1:33704–33,721	
	P: VIC-ACGCCTCTCAAGTGCCTACG- BHQ2	AB012956.1:33653–33,672	
*ctx*A	F: GGCTACAGAGATAGATATTACAG	AB699245.1:484–506	99
	R: TTCCCTCCAAGCTCTATG	AB699245.1:565–582	
	P: Cy5-CTGCCAATCCATAACCATCTGCTG- BHQ2	AB699245.1:527–550	

F, forward primer; R, reverse primer; P, Taqman probe; BHQ, black hole quencher. Reference seq ID: the accession No. of the sequence in NCBI GenBank.

### Quantitative real-time PCR

The singleplex real-time PCR reaction system targeting each gene consists of the following components: PerfeCTa qPCR ThoughMix (2×) (Apexbio, China) 10 μL, 0.4 μM primers, 0.2 μM probe, 2 μL of DNA, supplemented with DNase-free water to a total volume of 20 μL. The cycling parameters were performed as follows: 95°C for 5 min (pre-denaturation), followed by 40 cycles of 95°C for 15 s (denaturation), 58°C for 45 s (annealing and extension). The qPCR was performed using a CFX 96 thermal cycler (BioRad, USA), and the Ct value less than 35 was set as positive. The qPCR reaction was performed with three independent replicates.

### ddPCR

Each single-target ddPCR reaction contained 5 μL PerfeCTa qPCR ThoughMix (5×), 2.5 μL fluorescein sodium salt (1.0 μM), 1.0 μM primers, 0.25 μM probe, 2.0 μL DNA, and DNAase-free water to a final volume of 25 μL. The thermal cycling conditions were initiated at 94°C for 5 min, 45 cycles, including 94°C for 15 s and 58°C for 45 s. The triplex ddPCR reaction consisted of 5 μL PerfeCTa qPCR ThoughMix (5×), 2.5 μL fluorescein sodium salt (1.0 μM), 1.0 μM primers for each target, 0.25 μM *rfb* O1 and *rfb* O139 probe, 0.4 μM *ctx*A probe, 2 μL DNA, and DNAase-free water to a final volume of 25 μ L. The thermal cycling conditions were the same as single-target ddPCR.

All ddPCR reactions were performed using the Naica^®^ system (Stilla Technologies, France), allowing the detection of the *rfb* O1, *rfb* O139, and *ctx*A genes. The system performs digital PCR by partitioning the samples into arrays of up to 30,000 micro-droplets called droplet crystals using a microfluidic Sapphire Chip (Stilla Technologies, France) and two dedicated instruments (Geode and Prism 3). The instrument has three fluorescence channels (FAM, VIC, and Cy5), simultaneously carrying out three color fluorescence readings representing the number of positive microdroplets containing *rfb* O1, *rfb* O139, and *ctx*A genes. The ddPCR parameter optimization and assays were performed using three independent replicates.

### Optimization of PMA treatment conditions

Briefly, 20 mM PMA (Biotium, USA) was diluted with DNAase-free water to make a 1.0 mM working solution and stored at −20°C in the dark. The bacterial suspensions of 1.53 × 10^2^ CFU/mL and 1.53 × 10^5^ CFU/mL were heat killed at 95°C for 10 min to evaluate the minimum concentration of PMA required to inhibit DNA amplification from the dead bacteria completely. Then, 300 μL bacterial suspension was treated with a series of PMA working solutions (PMA final concentrations were 0, 2.5, 5, 10, 20, and 40 μM), followed by incubation in the dark for 15 min. The samples were exposed to a halogen lamp (650 W) on ice for 20 min. After DNA extraction, qPCR was performed using the reaction system and conditions mentioned above.

A high nucleic acid dye concentration may inhibit viable bacteria’s DNA amplification (Fittipaldi et al., 2012). 300 μL viable bacterial suspensions containing 1.53 × 10^2^ CFU/mL and 1.53 × 10^5^ CFU/mL were treated with a series PMA working concentration and performed by qPCR as described above to determine the maximum PMA concentration that did not produce this deviation.

The heat-killed cell suspension was tested at different light exposure times (0, 5, 10, 15, 20, 25, and 30 min) to identify the suitable exposure time at the optimal PMA concentration determined by the above experiments. PMA parameter optimization was carried out by three independent replicates.

### Sensitivity and specificity of PMA-triplex ddPCR

*V. cholerae* O1 and O139 gradient dilutions (1.53 × 10^6^ ~  1.53 × 10^1^ CFU/mL, signed as V6 ~ V1) were detected using PMA-triplex ddPCR and PMA-qPCR, respectively (Supplementary Table S2). *V. cholerae* genomic DNA (10.0 pg./μL) carrying *ctx*A virulence gene was diluted in a 10-fold gradient to 0.1 fg/μL in DNase-free water (signed as C6 ~ C1) and detected using PMA-triplex ddPCR and PMA-qPCR (Supplementary Table S3). According to NCCLS EP17-A guidelines, 30 DNAase-free water samples were tested using ddPCR with the Naica^®^ system as blank samples (Moretti et al., 2011) to determine the limit of blank (LOB) for each target. A minimum of five independently prepared Low-Level (LL) samples were detected with six replicates to determine the limit of detection (LOD). The LL samples used to calculate LOD were representative positive samples within a concentration range of one to five times higher than the calculated LOB.

Additionally, the method specificity was evaluated against 17 pathogens, including six non-O1/O139 *V. cholerae* strains and 11 other pathogens (*V. parahaemolyticus, L. monocytogenes, E. coli, Staphylococcus aureus, Enterobacter sakazakii, V. vulnificus, V. fluvialis, V. mimicus, Salmonella typhimurium, Shigella flexneri*, and *V. alginolyticus*). DNA samples were quantified to 0.1 ng/μL as ddPCR templates. Each DNA was detected three independent times.

### Enumeration of *Vibrio cholerae* at different proportions of viable bacteria

Briefly, the suspensions of heat-killed and live O1/O139 *V. cholerae* (both 1.53 10^5^ CFU/mL), were mixed in proportion to achieve a final concentration of live bacteria as 0, 5, 10, 25, 50, and 100% (Sanchez et al., 2013) (The total number of cells in the mixture was kept constant at 1.53 × 10^5^ CFU/mL), and 300 μL of the compound was taken after incubation at 37 °C for 5 min. Then, the mixtures were treated under the optimal conditions of PMA and subjected to ddPCR and qPCR. Three independent replicates were done for each test.

### Evaluation of quantitative detection of *Vibrio cholerae* in spiked seawater samples

Artificial seawater (ASW) was used to simulate seawater samples contaminated by *V. cholerae*. Sea salt (40 g/L; Sigma-Aldrich, Inc., St. Louis, USA) was dissolved in distilled water and then sterilized using a 0.22-μm membrane filter (Millipore, USA). After that, 4 mL aliquots of the ASW samples were inoculated with 1 mL of *V. cholerae* suspension at a final concentration of 1.53 × 10^2^ ~ 1.53 × 10^6^ CFU/mL to prepare spiked seawater samples, and 300 μL of the spiked seawater samples were used for PMA-ddPCR. LOBs and LODs of seawater samples were determined according to the NCCLS guidelines of EP17-A described above.

Furthermore, 25 simulated seawater samples were prepared for the evaluation of ddPCR tolerance to seawater, including 16 positive and nine negative samples (Supplementary Table S4). Among the positive samples, 12 were spiked with a single target, while four were spiked with three targets. The concentrations of *V. cholerae* O1 and O139 were 1.53 × 10^2^ and 1.53 × 10^5^ CFU/mL in the simulated seawater samples, respectively. All samples were tested using multiplex ddPCR and single-target qPCR. The ddPCR and qPCR were performed with three independent replicates.

### Statistical analysis

The LOBs and LODs at a 95% confidence level for ddPCR were automatically estimated using the online Gene-Pi statistical tool.^1^ SPSS 21.0 was used for statistical analysis. One-way ANOVA and Tukey’s t-test were employed for comparison among the groups, and Student’s t-test was used to compare two groups. Data were expressed as mean ± SD (standard deviation). *p* < 0.05 was considered to be statistically significant. GraphPad Prism 8.0 software was used for linear regression analysis and plotting, and each data was derived from triplicate measurements.

## Results

### The basic performance of qPCR, singleplex ddPCR, and triplex ddPCR

To establish a triplex digital PCR method, we first developed a singleplex ddPCR assay for each target. *V. cholerae* O1 (N16961) and O139 (MO45) genomic DNA were diluted according to a 10-fold gradient (3.0 fg/μL to 3.0 ng/μL) and detected using qPCR and singleplex ddPCR, respectively. Based on the results of these two methods, the triplex ddPCR for the simultaneous detection of three targets was successfully established through system optimization. The results revealed that the Ct value of each target detected using qPCR had a high linear relationship with the logarithm of DNA concentration (30.0 fg/μL to 3.0 ng/μL) (The linear fitting coefficient *R*^2^ = 0.995 ~ 0.998), while the detection value of each target tested by ddPCR had a linear relationship with the DNA concentration (3.0 fg/μL ~ 300 pg./μL) (*R*^2^ = 0.991 ~ 0.999). Triplex ddPCR and singleplex ddPCR results did not differ significantly (*p* > 0.05, Table 2). However, when the DNA concentration reached 3.0 ng/μL, ddPCR could not quantify because the number of positive droplets reached saturation.

**Table 2:** Quantitative analysis of *rfb* O1, *rfb* O139, and *ctx*A genes using qPCR, singleplex ddPCR, and triplex ddPCR ($\bar{X}$ ± SD).

qPCR (Ct)			
Genomic DNA	*rfb* O1	*rfb* O139	*ctx*A
3 ng/μL	17.35 ± 0.25	18.02 ± 0.28	17.78 ± 0.49
300 pg./μL	20.42 ± 0.33	21.69 ± 0.32	20.51 ± 0.39
30 pg./μL	24.09 ± 0.21	25.11 ± 0.48	23.11 ± 0.48
3 pg./μL	27.44 ± 0.34	28.41 ± 0.72	26.32 ± 0.29
300 fg/μL	30.79 ± 0.27	31.89 ± 0.67	29.66 ± 0.53
30 fg/μL	34.14 ± 0.29	34.83 ± 0.79	33.76 ± 0.42
3 fg/μL	36.75 ± 0.42	37.56 ± 0.83	35.87 ± 0.37
0.3 fg/μL	UD	UD	UD
*R* ^2^	0.998	0.998	0.995

ddPCR (copies/μL)						
Genomic DNA	Singleplex			Triplex		
	*rfb* O1	*rfb* O139	*ctx*A	*rfb* O1	*rfb* O139	*ctx*A
3 ng/μL	>ULOD	>ULOD	>ULOD	>ULOD	>ULOD	>ULOD
300 pg./μL	5724.31 ± 380.94	5068.67 ± 281.48	5482.33 ± 252.53	5647.66 ± 284.15	5240.78 ± 304.70	5343.33 ± 295.03
30 pg./μL	594.35 ± 16.26	441.65 ± 24.24	533.93 ± 28.88	581.68 ± 20.42	434.10 ± 19.53	543.93 ± 24.01
3 pg./μL	55.65 ± 5.59	47.83 ± 4.30	55.41 ± 7.57	52.65 ± 6.14	41.92 ± 5.62	51.03 ± 5.15
300 fg/μL	5.47 ± 0.52	4.68 ± 0.47	5.78 ± 0.47	5.26 ± 0.38	4.26 ± 0.33	6.48 ± 0.61
30 fg/μL	0.94 ± 0.09	0.39 ± 0.06	0.47 ± 0.13	0.91 ± 0.11	0.39 ± 0.05	0.92 ± 0.17
3 fg/μL	0.08 ± 0.02	0.12 ± 0.03	0.07 ± 0.02	0.09 ± 0.02	0.13 ± 0.02	0.08 ± 0.02
0.3 fg/μL	UD	UD	UD	UD	UD	UD
*R* ^2^	0.999	0.999	0.999	0.997	0.997	0.991

ULOD, upper limit of detection; UD, undetectable.

### Simultaneous detection of multiple targets using ddPCR

**Figure 1:**
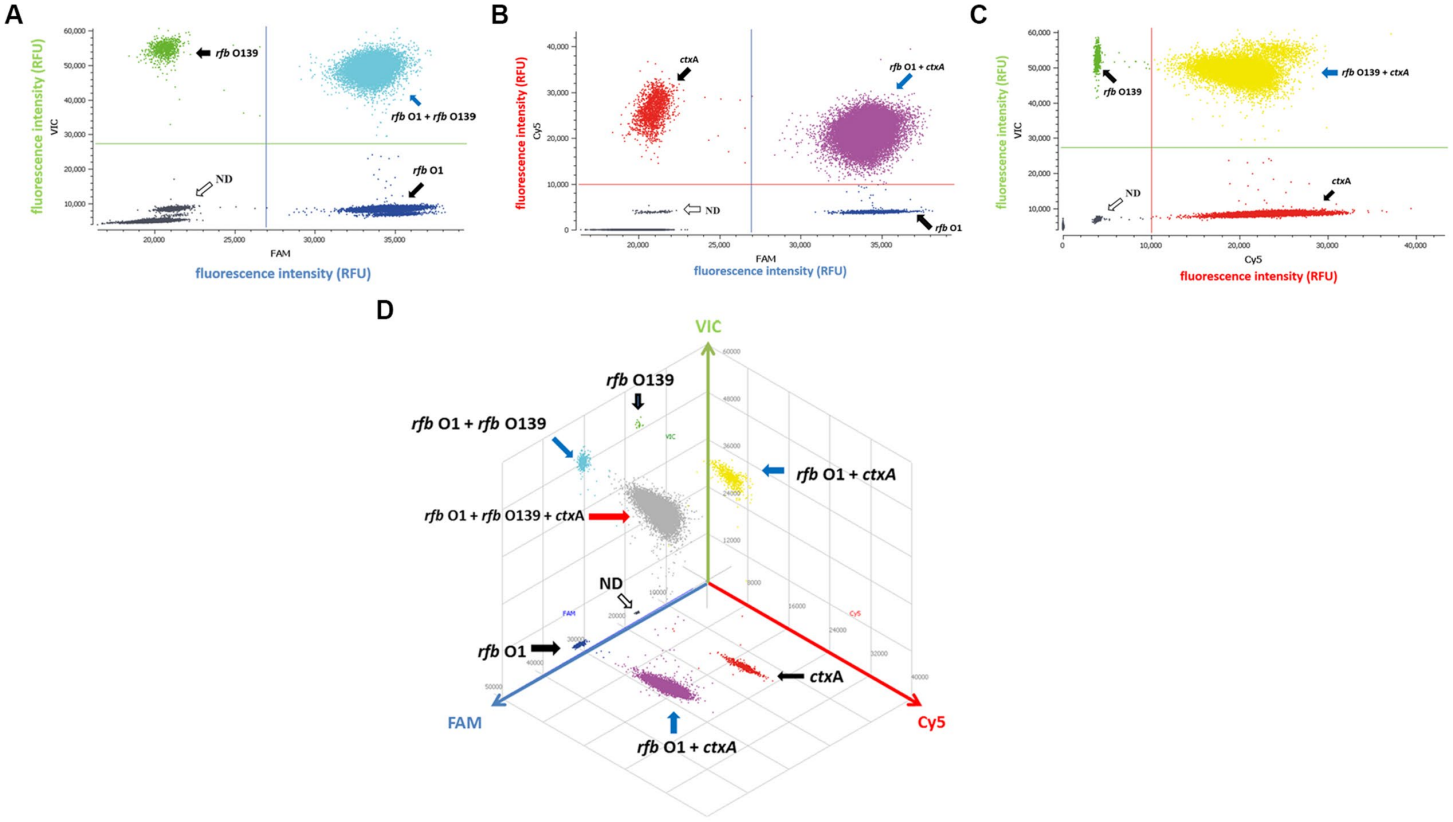
Simultaneous detection of two or three targets of *V. cholerae* using triplex ddPCR. **(A–C)** 2D scatter plot of the two targets detected using triplex ddPCR. In the 2D plot, the fluorescence intensity value for one of the three fluorescent channels is plotted onto the horizontal axis, while the fluorescence intensity value for one of the other two detection channels is plotted onto the vertical axis. **(D)** 3D scatter plot of the fluorescence intensities in the FAM acquisition channel (x axis), VIC acquisition channel (y axis) and Cy5 acquisition channel (z axis). The 8-color mode allows the fluorescence distribution in all three channels to be projected on 2D and 3D dot plots. For example, the population in **(A)** is composed of blue (+, −, −), green (−,+,−), cyan (−, −, +) and dark grey (−,−,−) dots, which represent FAM positive, VIC positive, both FAM and VIC positive, and negative, respectively. Droplet clusters marked by black, blue, and red arrows refer to positive droplets containing single, two, and three targets, respectively. ND: negative droplets without target genes.

Since a suspicious sample may contain multiple targets, we investigated the assay’s ability to detect two or three DNA targets simultaneously. When two targets were present in a sample, ddPCR produced 4 (2^2^) droplet clusters on the 2D scatter plot: one corresponding to the negative droplet with no target genes, another two corresponding to droplets with a single target, and the last one corresponding to a droplet cluster containing both genes (Figures 1A–C). When three targets were present in a sample, the 3D scatter plot revealed that the droplet cluster was divided into 8 (2^3^) clusters (Figure 1D). Each new cluster can be interpreted regarding its target type depending on the fluorescence intensity and location.

### The optimal concentration and light exposure time of PMA treatment

**Figure 2:**
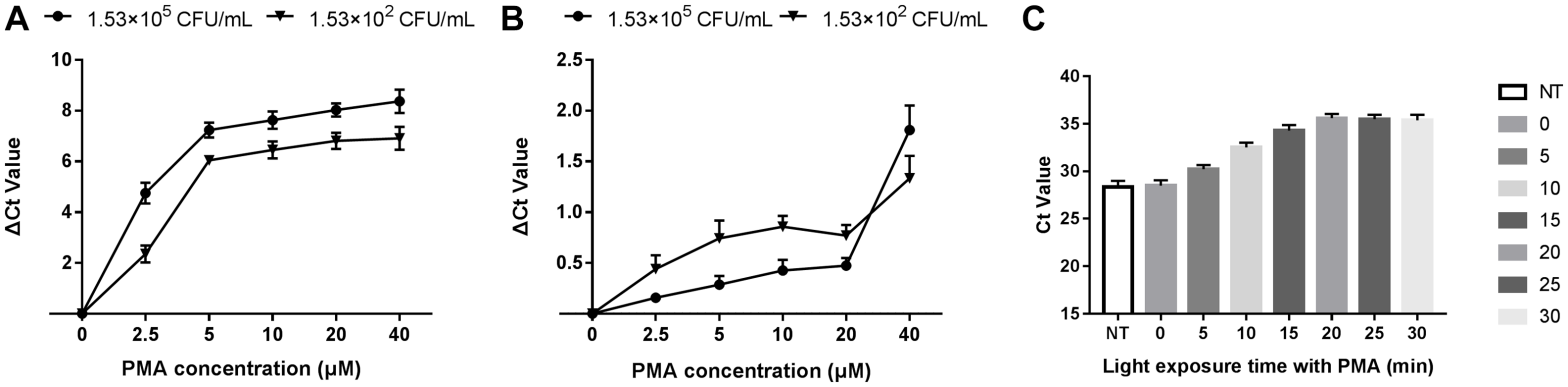
Optimization of PMA treatment conditions. **(A)** qPCR results of dead bacteria treated with different PMA concentrations. **(B)** qPCR results of live bacteria treated with different PMA concentrations. **(C)** qPCR results of dead bacteria after different times of light exposure. ΔCt = Ct value of the experimental group treated with PMA-Ct value of the control group at the same concentration without PMA treatment. NT: the control untreated with PMA.

The optimization of PMA treatment conditions mainly includes PMA concentration and exposure time. Figure 2A depicts that the ∆Ct value increased with the PMA concentration in the heat-killed bacteria group and entered the plateau at 20 μM, indicating that the inhibition effect of PMA on DNA amplification from dead bacteria increased with increasing PMA concentration. The minimum PMA concentration that completely inhibited the amplification of dead bacteria increased as the concentration of dead bacteria elevation. The ∆Ct values of different concentrations of dead bacteria were different under the same concentration of PMA treatment. The PMA concentration that completely inhibited the amplification of the lower concentration of dead bacteria was correspondingly lower. The two different concentrations of live bacteria were treated with a range of PMA concentrations followed by qPCR. Figure 2B reveals that when the PMA concentration did not exceed 20 μM, ΔCt values fluctuated from 0 to 0.69, indicating that these PMA concentrations did not significantly inhibit DNA amplification from viable bacteria. However, ΔCt values exceeded 1.0 when the PMA concentration was more than 20 μM, indicating that PMA had a significant inhibitory effect above this concentration. In this study, 20 μM was the optimal concentration of PMA treatment based on the results of PMA inhibiting the dead bacteria DNA amplification. Figure 2C displays the results of different light exposure time groups, which shows that the Ct values increased with the extension of light exposure time, reaching the highest value at 20 min, indicating that this light exposure time had the optimal inhibitory effect on *V. cholerae* DNA amplification. The results demonstrated that the optimal PMA treatment strategy involved a final PMA concentration of 20 μM and 20 min light exposure with halogen lamp at a level of 650 W.

### Performance of PMA-triplex ddPCR and PMA-qPCR to detect *Vibrio cholerae* under pure culture conditions

**Figure 3:**
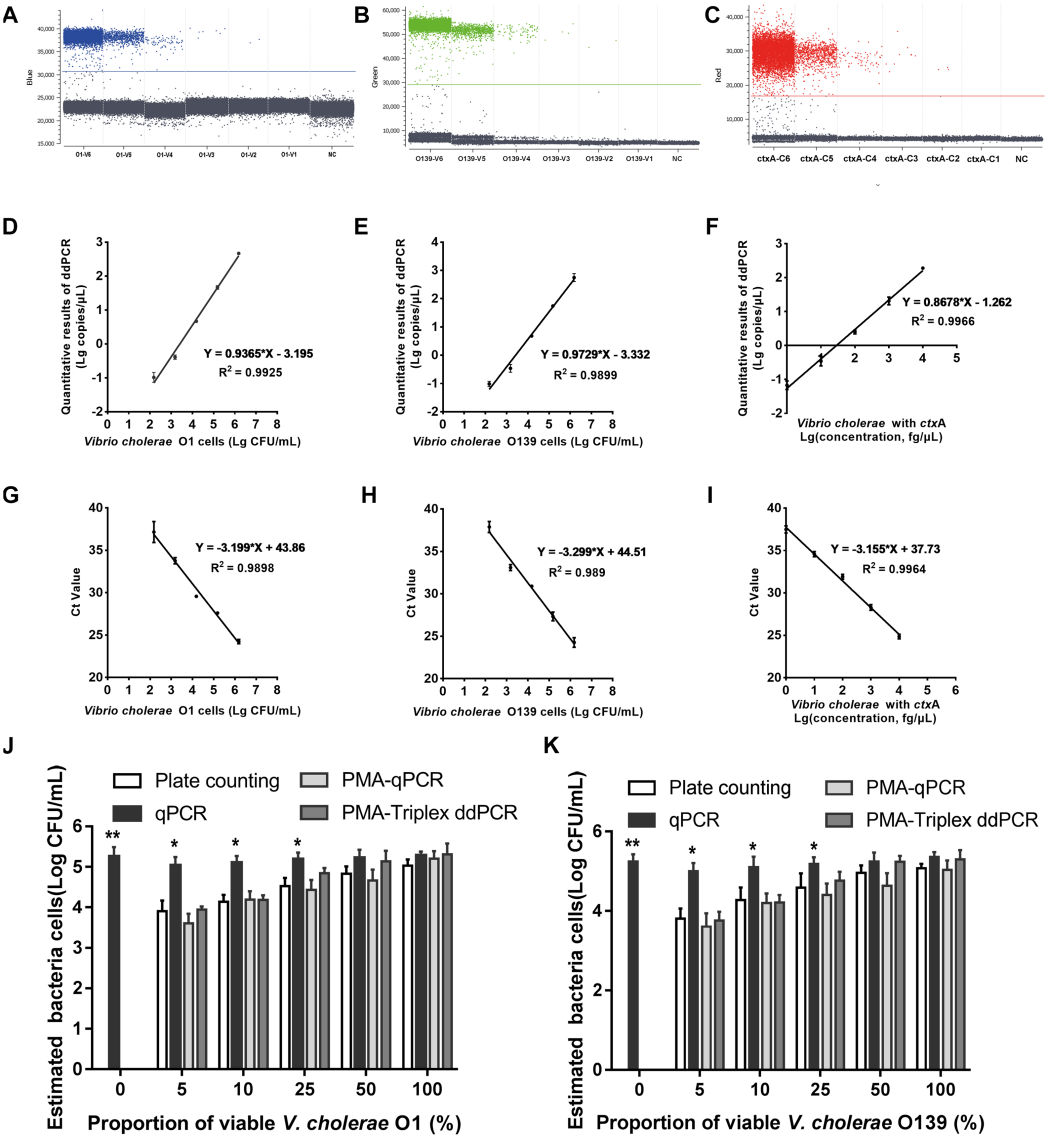
Performance of PMA-triplex ddPCR and PMA-qPCR for *V. cholerae* under pure culture conditions. **(A–C)** The 1D scatter plot of the *rfb O1* gene detected by FAM channel; The 1D scatter plot of *rfb O139* gene detected by VIC channel; The 1D scatter plot of the *ctx*A gene detected by CY5 channel. **(D–F)** Linear regression analysis based on PMA-triplex ddPCR in pure culture. **(G–I)** Linear regression analysis based on PMA-qPCR in pure culture. **(J,K)** Quantifying *V. cholerae* O1 and O139 at different proportions of viable bacteria using plate counting, qPCR, PMA-qPCR, and PMA-triplex ddPCR. Error bars indicate standard deviation of triplicate. **p < 0.01, *p < 0.05.

The 1D scatter plot of PMA-triplex ddPCR (Figures 3A,B) demonstrated that the number of positive droplets from V1 to V6 increased linearly with the rising bacterial concentrations. No positive droplet was detected in the negative control (NC), indicating no non-specific amplification in this system. The correlation coefficient *R*^2^ and regression equations of PMA-triplex ddPCR and PMA-qPCR were shown in Figures 3D,E,G,H. The LODs of the PMA-triplex ddPCR were estimated automatically at the 95% confidence level under pure culture conditions using the Gene-Pi online statistical tool. The LODs of PMA-triplex ddPCR for *V. cholerae* O1 and O139 were 127.91 and 120.23 CFU/mL, respectively, while PMA-qPCR sensitivity for O1 and O139 *V. cholerae* was estimated to be on the order of 10^3^ CFU/mL.

*Vibrio cholerae* genomic DNA *C6 ~ C1* carrying *ctx*A virulence gene was detected by PMA-triplex ddPCR and PMA-qPCR. The results revealed a high linear relationship (R^2^ ≥ 0.996) with the measured values ranging from 1.0 fg/μL to 10.0 pg./μL (Figures 3C,F,I; Supplementary Table S3). The LOD of *ctx*A detected by PMA-triplex ddPCR was as low as 1.5 copies/reaction (1.0 fg/μL) under pure culture conditions, while the sensitivity of PMA-qPCR was about 10.0 fg/μL.

The primers and probes produced specific amplification droplets for the respective DNA of positive strains. The absence of positive signals was detected in the blank and other negative strains, indicating that the primers and probes had good specificity (Supplementary Table S5).

### Validation of the ability of ddPCR to detect viable cells

The samples containing different viable *V. cholerae* proportions of (0, 5, 10, 25, 50, and 100%) were detected using plate counting, qPCR, PMA-qPCR, and PMA-ddPCR, respectively. Figures 3J,K indicate a significant difference between the qPCR and plate counting results. The qPCR results were unaffected by the proportion of viable bacteria, which is only related to the total amount of bacterial DNA. The results obtained by the PMA-triple ddPCR method were not significantly different from that of the plate counting method, indicating that this method could truly reflect the viable cell concentration of *V. cholerae*.

### Performance of PMA-ddPCR to detect spiked seawater samples

**Figure 4:**
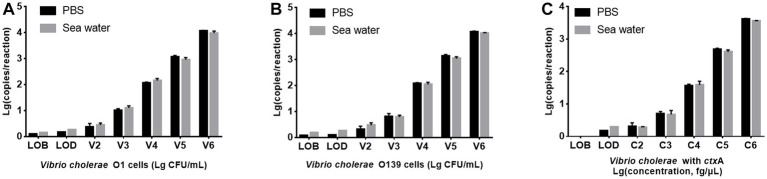
Comparison of PMA-triplex ddPCR for detecting *V. cholerae* in PBS solution and seawater samples. **(A,B)** The targets of *V. cholerae* O1 and O139, respectively. The x-axis represents Lg (bacterial concentration, CFU/mL), and the *y*-axis represents Lg (ddPCR results, copy number/reaction). **(C)** The targets of the *ctx*A gene. The *x*-axis represents Lg (DNA concentration, fg/μL), and the y-axis represents Lg (ddPCR results, copy number/reaction). Each test was repeated three times.

Seawater is a common environmental sample containing various PCR inhibitors (Ijzerman et al., 1997). *V. cholerae* O1 and O139 were added to artificial seawater to prepare seawater samples with the bacterial suspension concentration of 1.53 × 10^2^ ~ 1.53 × 10^6^ CFU/mL, respectively, then detected using PMA-triplex ddPCR to evaluate the system’s tolerance to seawater substrate. Figure 4 illustrates that *V. cholerae* O1 and O139 are lower in seawater samples than in PBS solution samples. However, LODs of *V. cholerae* O1 and O139 did not differ significantly between seawater samples and pure culture solutions (150.66 and 147.57 CFU/mL vs. 127.91 and 120.23 CFU/mL, *p* > 0.05). In contrast, LODs of *ctx*A did not differ significantly between seawater samples and pure culture solution (2 vs. 1.5 copies/reaction, *p* > 0.05). These results confirmed the applicability of PMA-triplex ddPCR for detecting the three targets in seawater samples.

As Supplementary Table S4 shown, among the 25 simulated seawater samples, 16 *V. cholerae* positive samples (1.53 × 10^2^ or 1.53 × 10^5^ CFU/mL double-target or triple-target *V. cholerae*) were identified as positive by PMA-triplex ddPCR with high sensitivity (100%, 16/16) and specificity (100%, 9/9). The single-target qPCR sensitivity was 50.0% (8/16) due to the inability to identify *V. cholerae* O1 and O139 at 1.53 × 10^2^ CFU/mL. The results revealed that the established PMA-triplex ddPCR method had higher sensitivity than PMA-qPCR and could be used to screen the threat of toxigenic *V. cholerae* O1 and O139 in suspicious seawater samples.

## Discussion

*Vibrio cholerae* is widely distributed in environmental water bodies. Regarding environmental monitoring, tools that provide quick and reliable results are essential for timely predicting ongoing or imminent cholera epidemic events, particularly during cholerae outbreaks (Ramamurthy et al., 2020). Rapid and sensitive multiplex analysis techniques have been proven to improve the detection efficiency of *V. cholerae* in suspicious samples. Several multiplex PCR-based methods, such as BD MAX (Becton Dickinson Inc., USA), have been developed for detecting O1, O139, and *ctx*A genes in feces (Li et al., 2021). Toxigenic and non-toxigenic *V. cholerae* were simultaneously quantified using real-time PCR in environmental water samples (Bliem et al., 2015), while virulence-related and species-specific genes of *V. cholerae* were detected using loop-mediated isothermal amplification (LAMP) assay (Xu et al., 2019). However, there have been few studies on ddPCR applications to detect *V. cholerae* in environmental water samples. Another concern is that viable pathogens are more responsible for health risks, and quantifying live cells is more valuable. Current mainstream technology for live cell detection combines PCR methods with biological dyes, such as ethidium monoazide (EMA) and propidium monoazide (PMA) (Nocker and Camper, 2006). This study established a method for simultaneous detection of *V. cholerae* O1/O139 antigen genes (*rfb* O1 and *rfb* O139) and enterotoxin gene (*ctx*A) established by combining triplex ddPCR with PMA treatment.

PMA is a more suitable dye for detecting food-borne pathogens in live cells than EMA, which has penetrated the damaged membranes of dead and viable cells, leading to false negative results in many studies. PMA-qPCR detection has been successfully applied to various foodborne pathogens (Zeng et al., 2016). Different species have different sensitivity to dye stress due to the different sensitivity and integrity of membranes. Therefore, DNA intercalation dyes should be optimized for each species (Fittipaldi et al., 2012). Proper PMA treatment conditions are critical for PMA-PCR because overtreatment can destroy live cells and lead to incorrect results (van Frankenhuyzen et al., 2011). In this study, we optimized PMA treatment conditions for *V. cholerae*, including appropriate PMA concentration and light exposure time. The samples were tested using qPCR after different treatment conditions, and the changes in Ct values were comprehensively evaluated. It was concluded that the best conditions to distinguish the live and dead *V. cholerae* cells were treated with 20 μM PMA in the dark for 15 min and exposed to a 650 nm halogen lamp for 20 min. The results were similar to Wu’s study (Wu et al., 2015), confirming that PMA treatment could distinguish between dead and live *V. cholerae*.

The technical breakthrough of microfluidic (Wu et al., 2015) has promoted the rapid development of digital PCR detection. Digital PCR, as a promising new nucleic acid detection technology, is bound to develop in the direction of multiple detections. The *rfb* O1 and *rfb* O139 genes are frequently used to distinguish the important serogroups of *V. cholerae*, whereas *ctx*A, which encodes cholera enterotoxin, is the most important determinant of virulence potential (Huang et al., 2009). These three target genes were suitable for simultaneously detecting two threatening endemic serogroups (O1 and O139) and their virulent capability. In this study, primers and probes of the three target genes were designed, and three fluorescence channels of the Naica Crystal digital PCR system were employed to meet actual monitoring requirements. In simulated seawater samples, the LODs of PMA-ddPCR for serogroups O1 and O139 were 150.66 and 147.57 CFU/mL, respectively, which were better than the qPCR sensitivity (on the order of 10^3^ CFU/mL). The method was also superior to the previous detection methods, such as 250 ~ 400 CFU/reaction of LAMP (Chen et al., 2022) and 1.0 × 10^4^ CFU/mL of monoclonal antibodies immunochromatography (Chen et al., 2014). However, when *V. cholerae* concentration is high (more than 10^7^ CFU/mL), producing excessive positive droplets, the DNA template must be diluted to avoid exceeding the ddPCR detection limit.

Another unique feature of digital PCR is that absolute quantification can be performed independently of standard curves (Li et al., 2018). In this study, the multiplex ddPCR was developed to target the single copy genes (*rfb* O1 and *rfb* O139) on *V. cholerae* chromosome. That is, the copy number correlated with the bacterial cell counts. The ddPCR detection results of the seawater samples demonstrated that the bacterial concentration obtained after conversion of the gene copy number was comparable to the plate counting results, indicating that the detection of single-copy genes by PMA-ddPCR was an efficient method for enumerating *V. cholerae* live cells.

Although the PMA-triplex ddPCR developed in this study has made some progress in detecting viable toxigenic *V. cholerae* O1 and O139, it still needs extensive repeated validation before its application. First, the environmental water samples’ quality complexity may affect the detection method’s accuracy (Li et al., 2015). For example, the difference in seawater salinity and turbidity level in different sea areas may affect the PMA treatment efficacy, resulting in the deviation of results. Second, the pathogen concentration is usually low in seawater. The traditional methods frequently require pretreatment steps, such as bacteria enrichment or proliferation, to improve the detection sensitivity. However, this will increase the workload and cannot standardize the quantitative results. The method developed in this study does not need to enrich and proliferate, thus reducing the workload. The method’s sensitivity will improve further once the pre-treatment steps are included. Third, there are problems with the efficiency of various methods for genome extraction, which may underestimate the actual contaminated level of *V. cholerae*. Recently, Minimum Information for Publication of Quantitative Digital PCR Experiments (dMIQE) guidelines (dMIQE Group, 2020) have been proposed and developed to solve these problems. The dMIQE adoption by researchers will help standardize experimental protocols, ensure rational technology development, maximize the efficient use of resources, and facilitate the widespread use of this powerful technology. However, some shortcomings need to be overcome for the popularization of digital PCR. Due to high instrument prices and usage costs, digital PCR is not yet widely equipped for cholera surveillance and control in field conditions, especially in resource-limited settings where advanced laboratory equipment may not be available. Portability and low cost may be the development tendency of digital PCR in the future.

## Conclusion

The rapid, sensitive, and accurate PMA-triplex ddPCR detection technique established in this study can be applied to the large-scale screening of toxigenic *V. cholerae* O1 and O139 in seawater samples, and is very useful for cholera epidemic prevention, control, and epidemiological monitoring.

## Data availability statement

The original contributions presented in the study are included in the article/Supplementary material, further inquiries can be directed to the corresponding author.

## Author contributions

JY: conceptualization, funding acquisition, and supervision. HX: methodology and writing original draft preparation. ZK: investigation. NK: validation and data curation. EZ: investigation and formal analysis. YQ: software. MH: visualization. All authors contributed to this article and approved the version final submitted.

## Funding

This work was sponsored by the Natural Science Foundation of Fujian Province (Grant No. 2022J01399) and Fujian Provincial health technology project (Grant No. 2020QNA021).

## Conflict of interest

The authors declare that the research was conducted in the absence of any commercial or financial relationships that could be construed as a potential conflict of interest.

## Publisher’s note

All claims expressed in this article are solely those of the authors and do not necessarily represent those of their affiliated organizations, or those of the publisher, the editors and the reviewers. Any product that may be evaluated in this article, or claim that may be made by its manufacturer, is not guaranteed or endorsed by the publisher.

## Footnotes

^1^ https://www.gene-pi.com/statistical-tools/lobLoD/
