# Supplementary material for: Absolute quantification of viable Vibrio cholerae in seawater samples using multiplex droplet digital PCR combined with propidium monoazide
Source: Front Microbiol. 2023 Jun 9;14:1149981. doi: 10.3389/fmicb.2023.1149981 (PMC10288211; doi:10.3389/fmicb.2023.1149981)
Supplement: Supplementary file 1 [file Data_Sheet_1.docx]

Supplementary Table S1 Information of the strains used in this experiment

| Strain code | Species | Source | Serogroup | *ctx*A |
| --- | --- | --- | --- | --- |
| N16961 | *Vibrio cholerae* | Our lab | O1 | + |
| MO45, ATCC 51394 | *Vibrio cholerae* | Our lab | O139 | + |
| FJ2007002 | *Vibrio cholerae* | Environmental isolate | O1 | - |
| FJ2021001 | *Vibrio cholerae* | Clinical isolate | O1 | + |
| FJ2009055 | *Vibrio cholerae* | Clinical isolate | O1 | - |
| FJ2008028 | *Vibrio cholerae* | Environmental isolate | O1 | + |
| FJ2017002 | *Vibrio cholerae* | Clinical isolate | O139 | + |
| FJ2008173 | *Vibrio cholerae* | Environmental isolate | O139 | - |
| FJ2022004 | *Vibrio cholerae* | Environmental isolate | O139 | + |
| FJ-SM6 | *Vibrio cholerae* | Clinical isolate | O139 | - |
| FJ2022001 | *Vibrio cholerae* | Clinical isolate | NOVC | + |
| FJ-H2017001 | *Vibrio cholerae* | Environmental isolate | NOVC | - |
| FJ-H0737 | *Vibrio cholerae* | Environmental isolate | NOVC | - |
| FJ-H0911 | *Vibrio cholerae* | Clinical isolate | NOVC | - |
| FJ-H2014046 | *Vibrio cholerae* | Clinical isolate | NOVC | - |
| FJ-H0915 | *Vibrio cholerae* | Environmental isolate | NOVC | - |
| FJ-H200827 | *Vibrio cholerae* | Environmental isolate | NOVC | - |
| FJ-H2008169 | *Vibrio cholerae* | Environmental isolate | NOVC | - |
| FJ-H200739 | *Vibrio cholerae* | Environmental isolate | NOVC | - |
| FJ-H201303 | *Vibrio cholerae* | Clinical isolate | NOVC | - |
| ATCC17802 | *Vibrio parahaemolyticus* | ATCC | / | / |
| ATCC 19115 | *L. monocytogenes* | ATCC | / | / |
| ATCC 25922 | *Escherichia coli* | ATCC | / | / |
| ATCC 6538 | *Staphylococcus aureus* | ATCC | / | / |
| ATCC 29544 | Enterobacter sakazakii | ATCC | / | / |
| ATCC 27562 | *Vibrio vulnificus* | ATCC | / | / |
| ATCC 33809 | *Vibrio Fluvialis* | ATCC | / | / |
| ATCC 33654 | *Vibrio mimicus* | ATCC | / | / |
| CMCC-B50115 | *Salmonella typhimurium* | CGMCC | / | / |
| CMCC-B51572 | *Shigella flexneri* | CGMCC | / | / |
| CMCC 1833 | *Vibrio alginolyticus* | CGMCC | / | / |

ATCC, American Type Culture Collection; CGMCC, China General Microbiological Culture Collection Center; NOVC, *V. cholerae* non-O1/non-O139; FJ, strains isolated from Fujian Province, China.

Supplementary Table S2 Comparison of PMA-triplex ddPCR and PMA-qPCR for detecting *V. cholerae* O1 and O139 under pure culture conditions

| Plate count  (CFU/mL) | PMA-qPCR (Ct) | |  | PMA- ddPCR (copies/µL) | |
| --- | --- | --- | --- | --- | --- |
|  | *rfb* O1 | *rfb* O139 |  | *rfb* O1 | *rfb* O139 |
| 1.53×10^6^ | 24.25±0.25 | 24.15±0.36 |  | 466.10±19.59 | 469.50±23.57 |
| 1.53×10^5^ | 27.61±0.11 | 27.55±0.28 |  | 46.34±5.96 | 55.36±6.79 |
| 1.53×10^4^ | 29.57±0.15 | 31.08± 0.21 |  | 4.65±0.32 | 4.84±0.29 |
| 1.53×10^3^ | 33.78±0.37 | 33.11±0.36 |  | 0.41±0.06 | 0.26±0.07 |
| 1.53×10^2^ | 37.16±1.21 | 37.37±0.63 |  | 0.11±0.03 | 0.08±0.02 |
| 1.53×10^1^ | UD | UD |  | 0 | 0 |

UD, undetectable.

Supplementary Table S3 Comparison of PMA-triplex ddPCR and PMA-qPCR for detecting *ctx*A gene under pure culture condition

| Genomic DNA | PMA-qPCR (Ct) | PMA- ddPCR (copies/µL) |
| --- | --- | --- |
| 10 pg/μL | 24.37±0.30 | 192.28±14.36 |
| 1 pg/μL | 27.84±0.37 | 21.16±5.45 |
| 100 fg/μL | 32.17±0.43 | 2.51±0.31 |
| 10 fg/μL | 34.54±0.63 | 0.36±0.11 |
| 1 fg/μL | 36.77±0.69 | 0.07±0.02 |
| 0.1 fg/μL | UD | UD |

UD, undetectable.

Supplementary Table S4: Comparison of PMA-triplex ddPCR and PMA-qPCR for detecting mixed target genes

| Sample  No. | Strains No. | Multiple targets | *V. cholerae*  (CFU/mL) | PMA- triplex ddPCR (copies/µL) | | |  | PMA-qPCR (Ct) | | |
| --- | --- | --- | --- | --- | --- | --- | --- | --- | --- | --- |
|  |  |  |  | *rfb* O1 | *rfb* O139 | *ctx*A |  | *rfb* O1 | *rfb* O139 | *ctx*A |
| 1 | N16961 | *rfb* O1, *ctx*A | 1.53×10^5^ | 42.24±4.12 | -/-/- | 52.18±6.79 |  | 27.48±0.15 | **-/-/-** | 27.13±0.17 |
| 2 | FJ2021001 | *rfb* O1, *ctx*A | 1.53×10^5^ | 49.51±3.75 | -/-/- | 57.23±4.83 |  | 27.79±0.23 | **-/-/-** | 27.63±0.26 |
| 3 | N16961 | *rfb* O1, *ctx*A | 1.53×10^2^ | 0.09±0.02 | -/-/- | 0.11±0.03 |  | 37.26±0.35 | **-/-/-** | 36.89±0.28 |
| 4 | FJ2008028 | *rfb* O1, *ctx*A | 1.53×10^2^ | 0.8±0.03 | -/-/- | 0.10±0.02 |  | 37.49±0.42 | **-/-/-** | 37.02±0.63 |
| 5 | MO45 | *rfb* O139, *ctx*A | 1.53×10^5^ | -/-/- | 51.43±5.36 | 56.77±6.82 |  | **-/-/-** | 27.34±0.27 | 27.20±0.32 |
| 6 | FJ2017002 | *rfb* O139, *ctx*A | 1.53×10^5^ | -/-/- | +/+/+ | +/+/+ |  | **-/-/-** | 28.14±0.37 | 27.99±0.31 |
| 7 | MO45 | *rfb* O139, *ctx*A | 1.53×10^2^ | -/-/- | 0.08±0.02 | 0.09±0.02 |  | **-/-/-** | 38.17±0.46 | 38.02±0.52 |
| 8 | FJ2022004 | *rfb* O139, *ctx*A | 1.53×10^2^ | -/-/- | 0.10±0.03 | 0.12±0.03 |  | **-/-/-** | 37.92±0.54 | 37.83±0.47 |
| 9 | FJ2009055  FJ2008173 | *rfb* O1  *rfb* O139 | 1.53×10^5^ | 49.87±5.72 | 53.64±6.17 | -/-/- |  | 27.64±0.36 | 27.33±0.45 | **-/-/-** |
|  |  |  |  |  |  |  |  |  |  |  |
| 10 | FJ2007002 FJ-SM6 | *rfb* O1  *rfb* O139 | 1.53×10^5^ | 51.22±6.89 | 54.37±7.28 | -/-/- |  | 27.86±0.52 | 27.53±0.37 | **-/-/-** |
|  |  |  |  |  |  |  |  |  |  |  |
| 11 | FJ2009055 | *rfb* O1 | 1.53×10^2^ | 0.07±0.02 | 0.10±0.03 | -/-/- |  | 37.97±0.44 | 37.62±0.59 | **-/-/-** |
|  | FJ2008173 | *rfb* O139 |  |  |  |  |  |  |  |  |
| 12 | FJ2007002 FJ-SM6 | *rfb* O1  *rfb* O139 | 1.53×10^2^ | 0.08±0.03 | 0.07±0.02 | -/-/- |  | 37.66±0.49 | 38.15±0.52 | **-/-/-** |
| 13 | N16961 | *rfb* O1, *ctx*A | 1.53×10^5^ | 48.25±5.32 | 51.67±5.12 | 57.31±6.76 |  | 27.56±0.32 | 27.33±0.43 | 27.07±0.24 |
|  | MO45 | *rfb* O139 |  |  |  |  |  |  |  |  |
| 14 | FJ2008028 FJ2008173 | *rfb* O1, *ctx*A  *rfb* O139 | 1.53×10^5^ | 50.12±6.56 | 47.96±5.167 | 53.21±5.80 |  | 27.40±0.27 | 27.81±0.35 | 27.21±0.41 |
| 15 | N16961  MO45 | *rfb* O1, *ctx*A  *rfb* O139 | 1.53×10^2^ | 0.08±0.02 | 0.07±0.02 | 0.11±0.03 |  | 37.86±0.52 | 38.07±0.63 | 37.52±0.45 |
| 16 | FJ2007002 FJ2022004 | *rfb* O1  *rfb* O139, *ctx*A | 1.53×10^2^ | 0.09±0.03 | 0.07±0.02 | 0.12±0.04 |  | 37.69±0.37 | 37.85±0.58 | 37.36±0.42 |
| 17 | FJ-H2017001 | NOVC | 1.53×10^5^ | -/-/- | -/-/- | -/-/- |  | **-/-/-** | **-/-/-** | **-/-/-** |
| 18 | FJ-H0737 | NOVC | 1.53×10^5^ | -/-/- | -/-/- | -/-/- |  | **-/-/-** | **-/-/-** | **-/-/-** |
| 19 | FJ-H0911 | NOVC | 1.53×10^5^ | -/-/- | -/-/- | -/-/- |  | **-/-/-** | **-/-/-** | **-/-/-** |
| 20 | FJ-H2014046 | NOVC | 1.53×10^5^ | -/-/- | -/-/- | -/-/- |  | **-/-/-** | **-/-/-** | **-/-/-** |
| 21 | FJ-H0915 | NOVC | 1.53×10^5^ | -/-/- | -/-/- | -/-/- |  | **-/-/-** | **-/-/-** | **-/-/-** |
| 22 | FJ-H200827 | NOVC | 1.53×10^5^ | -/-/- | -/-/- | -/-/- |  | **-/-/-** | **-/-/-** | **-/-/-** |
| 23 | FJ-H2008169 | NOVC | 1.53×10^5^ | -/-/- | -/-/- | -/-/- |  | **-/-/-** | **-/-/-** | **-/-/-** |
| 24 | FJ-H200739 | NOVC | 1.53×10^5^ | -/-/- | -/-/- | -/-/- |  | **-/-/-** | **-/-/-** | **-/-/-** |
| 25 | FJ-H201303 | NOVC | 1.53×10^5^ | -/-/- | -/-/- | -/-/- |  | **-/-/-** | **-/-/-** | **-/-/-** |

NOVC, *V. cholerae* non-O1/non-O139; “-/-/- “means that all three replicates were negative.

Supplementary Table S5 Detection of specificity for PMA-ddPCR

| NO. | Strain code | Species | Serogroup | *ctx*A | ddPCR |
| --- | --- | --- | --- | --- | --- |
| 1 | FJ-H2014046 | *Vibrio cholerae* | NOVC | **-** | **-/-/-** |
| 2 | FJ-H0915 | *Vibrio cholerae* | NOVC | **-** | **-/-/-** |
| 3 | FJ-H200827 | *Vibrio cholerae* | NOVC | **-** | **-/-/-** |
| 4 | FJ-H2008169 | *Vibrio cholerae* | NOVC | **-** | **-/-/-** |
| 5 | FJ-H200739 | *Vibrio cholerae* | NOVC | **-** | **-/-/-** |
| 6 | FJ-H201303 | *Vibrio cholerae* | NOVC | **-** | **-/-/-** |
| 7 | ATCC17802 | *Vibrio parahaemolyticus* | / | / | **-/-/-** |
| 8 | ATCC 19115 | *L. monocytogenes* | / | / | **-/-/-** |
| 9 | ATCC 25922 | *Escherichia coli* | / | / | **-/-/-** |
| 10 | ATCC 6538 | *Staphylococcus aureus* | / | / | **-/-/-** |
| 11 | ATCC 29544 | Enterobacter sakazakii | / | / | **-/-/-** |
| 12 | ATCC 27562 | *Vibrio vulnificus* | / | / | **-/-/-** |
| 13 | ATCC 33809 | *Vibrio Fluvialis* | / | / | **-/-/-** |
| 14 | ATCC 33654 | *Vibrio mimicus* | / | / | **-/-/-** |
| 15 | CMCC-B50115 | *Salmonella typhimurium* | / | / | **-/-/-** |
| 16 | CMCC-B51572 | *Shigella flexneri* | / | / | **-/-/-** |
| 17 | CMCC 1833 | *Vibrio alginolyticus* | / | / | **-/-/-** |

NOVC, *V. cholerae* non-O1/non-O139; **“-/-/-“** means that all three replicates were negative
